# Fluctuation in SARS-CoV-2 Environmental Surface Contamination Levels in Homes Where Patients With COVID-19 Stayed for Recuperation

**DOI:** 10.7759/cureus.52055

**Published:** 2024-01-10

**Authors:** Hidehito Matsui, Chihiro Ueda, Eri Nakajima, Yukiko Takarabe, Yukie Yamaguchi, Yumiko Suzuki, Harumi Endo, Miho Sugamata, Yasuo Imoto, Hideaki Hanaki

**Affiliations:** 1 Research Center for Infection Control, Ōmura Satoshi Memorial Institute, Kitasato University, Tokyo, JPN; 2 R&D Team, Kobe Testing Center, The Japan Textile Products Quality and Technology Center, Hyogo, JPN

**Keywords:** fomites, housebound patient, environmental contamination, sars-cov-2, covid-19

## Abstract

Introduction

Transmission of severe acute respiratory syndrome coronavirus 2 (SARS-CoV-2) often occurs among family members. Elucidating where viable SARS-CoV-2 virions, and not just residual viral RNA, are present in the house is necessary to prevent the further spread of the coronavirus disease 2019 (COVID-19). We aimed to evaluate the environmental surface contamination levels of both SARS-CoV-2 RNA and viable viruses in the homes of housebound patients with COVID-19.

Methods

Environmental samples were collected from the households of three patients in April and July 2022 when the number of new COVID-19 cases in Japan was reported to be approximately 50,000 and 200,000 cases per day, respectively. For each case, samples were obtained from 19-26 household sites for seven consecutive days. SARS-CoV-2 RNA was examined in 455 samples through reverse transcription-quantitative polymerase chain reaction (RT-qPCR), and RT-qPCR-positive samples were subjected to plaque assay to detect viable viruses.

Results

Among the 455 samples, 63 (13.8%) that were collected from patients’ pillows and comforters, doorknobs, chairs, and refrigerators tested positive by RT-qPCR. The maximum detection rate of SARS-CoV-2 RNA-positive samples in each case ranged from 20.0% to 57.7% on days 1 to 3. The detection rate gradually decreased to 0-5.3% as the days elapsed. Although all RT-qPCR-positive samples were examined, no viable viruses were detected in these samples.

Conclusions

Although environmental contamination of SARS-CoV-2 RNA was observed in the homes of housebound patients with COVID-19, no viable viruses were isolated. This suggests that the indirect transmission risk from fomites was low.

## Introduction

The coronavirus disease 2019 (COVID-19) pandemic has inflicted substantial damage on public health and economic activity. Severe acute respiratory syndrome coronavirus 2 (SARS-CoV-2), the causative agent of COVID-19, has rapidly spread worldwide, with viral genomic mutations enabling the emergence of variant strains [1,2]. Although the Omicron variant, which was first isolated in late 2021 in South Africa, has higher transmissibility than the Delta variant and has spread worldwide [3,4], the COVID-19 severity caused by the Omicron variant was lower than that caused by the Delta variant. In a cohort study conducted in England, the adjusted hazard ratio of hospital admission and death caused by the Omicron variant infection compared with the Delta variant infection were 0.41 or 0.31, respectively [5]. In surveillance data from the USA, the infection fatality ratio caused by the Omicron variant was approximately five-fold lower than that of the Delta variant [6]. Moreover, the provision of vaccinations, the establishment of medical care systems and clinical practice guidelines for COVID-19, and the development of novel antiviral agents and neutralizing monoclonal antibodies have reduced the mortality rates and infection severity. Thus, the number of patients with mild COVID-19 has gradually increased, and almost all infected patients with no risk factors were quarantined in their homes or recovery accommodation facilities for COVID-19. The secondary attack rates of the Omicron variant in households were 25.1% and 29.0% in studies conducted in Denmark and Norway, respectively [7,8]. Therefore, infection control for household transmission of SARS-CoV-2 is important for reducing the risk of secondary attacks. Although the known routes of SARS-CoV-2 transmission to humans are droplet, aerosol, and contact infections [9,10], the environmental contamination level of SARS-CoV-2 during home quarantine remains unclear. Therefore, we aimed to investigate the fluctuation in environmental surface contamination levels of SARS-CoV-2 and the presence of viable viruses using quantitative reverse transcription polymerase chain reaction (RT-qPCR) and the plaque assay method in the homes of housebound patients with COVID-19.

## Materials and methods

Collection of environmental samples from the homes of patients with COVID-19

Suitable sample collection is an important factor for accurate data analysis. Therefore, the homes of microbiology researchers who also have healthcare professional licenses were targeted if a family member with COVID-19 stayed there for recuperation. Moreover, to further evaluate the temporal changes in environmental contamination, only the cases where sample collection over seven consecutive days by microbiology researchers was possible were ultimately included in this study. Based on these criteria, we included three cases of environmental sample collection from the homes of housebound patients with COVID-19 who tested positive by rapid antigen test for SARS-CoV-2.

Environmental sample collection was performed in April and July 2022 in the homes of patients residing in the Kanto region of Japan (Table 1). In all cases, environmental samples from the patient's home were collected for seven consecutive days by one of the family members, a different microbiology researcher in each case. Day 0 was defined as the day when a positive result by rapid antigen test was obtained. Details of the sampled objects are presented in Tables 2-4. The target object surfaces (5 cm × 5 cm) were rubbed using a sterile polyester swab (JAPAN COTTON BUDS Industry Limited, Tokyo, Japan) longitudinally and laterally 10 times for the collection of each sample. In the sites where samples were difficult to collect using a 5 cm × 5 cm square area because of the object shape, sampling was carried out in a similar dimension. The collected swab samples were suspended in 1.5 mL of universal transport medium (COPAN Diagnostics, Murrieta, CA, USA) and transported to the laboratory at temperatures under 10°C. The samples were then stored at −80°C until further use. Because we did not collect treatment information and individual patient samples, this study was exempted from the ethical regulations in Japan.

**Table 1:** Characteristics of the three homes used for recuperation

	Case 1	Case 2	Case 3
House location	Kanagawa	Kanagawa	Saitama
Date of sampling	April 2022	April 2022	July 2022
House type	Condominium	Detached house	Detached house
Family members			
Male	2	2	2
Female	2	2	2
Antigen test positive member	1	1	1
Bedrooms in house	4	4	3
Sampling sites per day	20	19	26

**Table 2:** RT-qPCR detection of SARS-CoV-2 RNA from environmental surface samples for case 1 Abbreviations: N.D., not detected; RT-qPCR, reverse transcription-quantitative polymerase chain reaction; SARS-CoV-2, severe acute respiratory syndrome coronavirus 2; RNA, ribonucleic acid

Sampled objects	Gene copy number in samples (copies/mL)						
	Day 1	Day 2	Day 3	Day 4	Day 5	Day 6	Day 7
Patient’s bedroom							
Pillow	N.D.	6.0×10^4^	N.D.	N.D.	N.D.	N.D.	N.D.
Comforter	1.5×10^5^	N.D.	N.D.	N.D.	N.D.	N.D.	N.D.
Sheet	N.D.	N.D.	N.D.	1.9×10^5^	N.D.	1.2×10^6^	N.D.
Bed rail	N.D.	N.D.	N.D.	N.D.	N.D.	N.D.	N.D.
Doorknob	N.D.	8.1×10^4^	N.D.	2.9×10^5^	N.D.	3.2×10^4^	N.D.
Mobile phone	1.0×10^5^	N.D.	2.9×10^5^	N.D.	N.D.	N.D.	N.D.
Computer keyboard	N.D.	N.D.	4.0×10^5^	N.D.	N.D.	1.6×10^5^	N.D.
Computer mouse	N.D.	N.D.	N.D.	N.D.	N.D.	N.D.	N.D.
Living room							
Sofa	2.4×10^5^	3.9×10^5^	N.D.	N.D.	N.D.	N.D.	N.D.
Table	N.D.	N.D.	N.D.	N.D.	N.D.	N.D.	N.D.
Chair	N.D.	N.D.	N.D.	N.D.	N.D.	N.D.	N.D.
Floor	N.D.	N.D.	N.D.	N.D.	N.D.	N.D.	N.D.
Bathroom							
Floor	N.D.	5.6×10^4^	N.D.	N.D.	N.D.	N.D.	N.D.
Doorknob	N.D.	N.D.	N.D.	N.D.	N.D.	N.D.	N.D.
Toilet seat	N.D.	N.D.	N.D.	N.D.	N.D.	N.D.	N.D.
Lavatory tap	N.D.	N.D.	N.D.	N.D.	N.D.	N.D.	8.3×10^4^
Lavatory bowl	N.D.	N.D.	N.D.	N.D.	N.D.	N.D.	N.D.
Bathtub drain	N.D.	N.D.	N.D.	N.D.	N.D.	N.D.	N.D.
Other							
Entrance doorknob	9.3×10^4^	N.D.	N.D.	N.D.	N.D.	N.D.	N.D.
Patient’s toothbrush	N.D.	N.D.	N.D.	N.D.	N.D.	N.D.	N.D.
Total positive sample no. (%)	4 (20.0)	4 (20.0)	2 (10.0)	2 (10.0)	0 (0.0)	3 (15.0)	1 (5.0)

**Table 3:** RT-qPCR detection of SARS-CoV-2 RNA in the environmental surface samples for case 2 Abbreviations: N.D., not detected; RT-qPCR, reverse transcription-quantitative polymerase chain reaction; SARS-CoV-2, severe acute respiratory syndrome coronavirus 2; RNA, ribonucleic acid

Sampled objects	Gene copy number in sample (copies/mL)						
	Day 2	Day 3	Day 4	Day 5	Day 6	Day 7	Day 8
Patient’s bedroom							
Pillow	N.D.	2.4×10^5^	N.D.	N.D.	5.7×10^4^	N.D.	N.D.
Comforter	1.8×10^5^	4.6×10^5^	N.D.	N.D.	N.D.	N.D.	N.D.
Sheet	N.D.	N.D.	1.5×10^5^	7.3×10^4^	N.D.	N.D.	N.D.
Kitchen, dining room, and living room							
Doorknob	N.D.	1.2×10^5^	N.D.	4.6×10^5^	6.0×10^4^	N.D.	N.D.
Backrest of chair	N.D.	6.9×10^4^	N.D.	N.D.	N.D.	N.D.	N.D.
Refrigerator	N.D.	8.2×10^4^	N.D.	N.D.	N.D.	N.D.	N.D.
Table	N.D.	N.D.	N.D.	N.D.	N.D.	2.8×10^4^	N.D.
Cupboard	N.D.	N.D.	N.D.	N.D.	N.D.	N.D.	N.D.
Remote controller for television	N.D.	N.D.	N.D.	N.D.	N.D.	N.D.	N.D.
Remote controller for air conditioner	N.D.	N.D.	N.D.	N.D.	N.D.	N.D.	N.D.
Bathroom							
Lavatory tap	N.D.	2.2×10^5^	N.D.	N.D.	N.D.	N.D.	N.D.
Floor	N.D.	N.D.	N.D.	N.D.	3.7×10^4^	N.D.	N.D.
Doorknob	N.D.	N.D.	N.D.	N.D.	N.D.	N.D.	N.D.
Toilet seat	N.D.	N.D.	N.D.	N.D.	N.D.	N.D.	N.D.
Room light switch	N.D.	N.D.	N.D.	N.D.	N.D.	N.D.	N.D.
Other							
Entrance doorknob	N.D.	N.D.	N.D.	N.D.	N.D.	N.D.	7.7×10^4^
Laptop keyboard	N.D.	N.D.	N.D.	N.D.	N.D.	N.D.	N.D.
Computer mouse	N.D.	N.D.	N.D.	N.D.	N.D.	N.D.	N.D.
Bedroom doorknob	N.D.	N.D.	N.D.	N.D.	N.D.	N.D.	N.D.
Total positive sample no. (%)	1 (5.3)	6 (31.6)	1 (5.3)	2 (10.5)	3(15.8)	1 (5.3)	1 (5.3)

**Table 4:** RT-qPCR detection of SARS-CoV-2 RNA in the environmental surface samples for case 3 Abbreviations: N.D.; not detected; RT-qPCR, reverse transcription-quantitative polymerase chain reaction; SARS-CoV-2, severe acute respiratory syndrome coronavirus 2; RNA, ribonucleic acid

Sampled objects	Gene copy number in sample (copies/mL)						
	Day 3	Day 4	Day 5	Day 6	Day 7	Day 8	Day 9
Patient’s bedroom							
Pillow	2.4×10^4^	1.3×10^5^	5.2×10^5^	N.D.	N.D.	4.4×10^4^	N.D.
Comforter	2.1×10^5^	N.D.	N.D.	N.D.	N.D.	N.D.	N.D.
Doorknob	1.7×10^4^	N.D.	N.D.	N.D.	N.D.	N.D.	N.D.
Kitchen and dining room							
Table	N.D.	N.D.	N.D.	N.D.	N.D.	5.0×10^5^	N.D.
Backrest of chair	2.4×10^4^	N.D.	N.D.	N.D.	7.1×10^4^	N.D.	N.D.
Refrigerator	6.3×10^4^	N.D.	N.D.	N.D.	6.4×10^4^	N.D.	N.D.
Cupboard	7.1×10^5^	N.D.	N.D.	N.D.	N.D.	N.D.	N.D.
Living room							
Doorknob	5.6×10^4^	N.D.	N.D.	N.D.	N.D.	N.D.	N.D.
Room light switch	3.2×10^4^	N.D.	N.D.	N.D.	N.D.	2.4×10^5^	N.D.
Child safety fence	N.D.	N.D.	N.D.	N.D.	N.D.	N.D.	N.D.
Remote controller for television	5.5×10^4^	N.D.	N.D.	N.D.	N.D.	4.2×10^5^	N.D.
Remote controller for air conditioner	6.4×10^4^	N.D.	N.D.	N.D.	N.D.	2.5×10^5^	N.D.
Air purifier (suction opening)	N.D.	6.9×10^5^	N.D.	N.D.	N.D.	N.D.	N.D.
Air purifier (discharge opening)	N.D.	N.D.	2.4×10^4^	N.D.	N.D.	N.D.	N.D.
Bathroom							
Room light switch	6.5×10^4^	7.0×10^5^	N.D.	N.D.	N.D.	N.D.	N.D.
Doorknob	2.5×10^4^	N.D.	N.D.	N.D.	N.D.	N.D.	N.D.
Lavatory bowl	8.0×10^4^	6.1×10^5^	N.D.	N.D.	N.D.	N.D.	N.D.
Lavatory tap	N.D.	N.D.	N.D.	N.D.	N.D.	N.D.	N.D.
Toilet seat	N.D.	N.D.	N.D.	N.D.	N.D.	N.D.	N.D.
Floor	N.D.	N.D.	N.D.	N.D.	N.D.	N.D.	N.D.
Other							
Laptop keyboard	1.1×10^6^	2.2×10^6^	2.6×10^6^	N.D.	N.D.	N.D.	N.D.
Pacifier	2.1×10^4^	N.D.	N.D.	1.9×10^4^	N.D.	N.D.	N.D.
Toy	N.D.	7.0×10^5^	N.D.	N.D.	N.D.	N.D.	N.D.
Plushie	N.D.	N.D.	N.D.	N.D.	N.D.	N.D.	N.D.
Picture book	N.D.	N.D.	N.D.	N.D.	N.D.	N.D.	N.D.
Mobile phone	N.D.	N.D.	N.D.	N.D.	N.D.	N.D.	N.D.
Total positive sample no. (%)	15 (57.7)	6 (23.1)	3 (11.5)	1 (3.8)	2 (7.7)	5 (19.2)	0 (0.0)

Detection of SARS-CoV-2 from environmental samples by quantitative reverse transcription polymerase chain reaction

The SARS-CoV-2 RNA was detected in the samples by RT-qPCR using a SARS-CoV-2 N2 gene detection kit (TOYOBO Co., Ltd., Osaka, Japan). The collected swab-suspended universal transport mediums were then diluted 10-fold with phosphate-buffered saline (Takara Bio Inc., Shiga, Japan) to avoid inhibition of the detection reaction by the medium components. A pre-treatment step was then performed by mixing 6 µL of a 10-fold-diluted medium sample and 3 µL of pre-treatment solution, and the mixture was heated at 95°C for 5 minutes to extract the target RNA. The following steps were performed according to the manufacturer’s protocol, and the amplified samples that were detected by the FAM channel within 45 cycles were considered positive for SARS-CoV-2. Finally, the SARS-CoV-2-positive control RNA (Nihon Gene Research Laboratories, Inc., Miyagi, Japan) was used for the standard curve, ranging from 10^4^ to 10^6^ copies/mL, allowing for the copy numbers of the target genes to be calculated.

Plaque assay for viable SARS-CoV-2

RT-qPCR-positive environmental samples were subjected to a plaque assay to detect viable SARS-CoV-2. VeroE6/transmembrane serine protease 2 (TMPRSS2) cells (JCRB1819) (JCRB Cell Bank, Osaka, Japan) were cultured in Dulbecco’s modified Eagle’s medium (DMEM) with low glucose (Sigma-Aldrich Japan, Tokyo, Japan) containing 5% fetal bovine serum (FBS) (Nichirei Biosciences Inc., Tokyo, Japan) and 1 mg/mL G418 (Fujifilm Wako Pure Chemical Corp., Osaka, Japan) at 37°C and 5% CO_2_ until sub-confluence was reached. The cultured cells were seeded in six-well plates at 6.5 × 10^5^ cells/well and incubated for three days at 37°C with 5% CO_2_ to form cell monolayers. The environmental samples were filtered using a 0.22-µm pore size membrane, and 0.1 mL of each sample was added to each of the 10 wells. After 1.5 hours of incubation at 37°C with 5% CO_2_, 2 mL of overlay medium containing 2% FBS and 0.01% diethylaminoethyl dextran in DMEM was added. After incubation for five days, the VeroE6/TMPRSS2 cells in the wells were fixed with 1% glutaraldehyde solution for 1 hour and stained with 0.0375% methylene blue for 20 minutes. The methylene blue solution was removed, and the samples were washed twice with water to count the plaques.

## Results

The homes of three patients with COVID-19 were included in this study. Samples were collected from two houses located in Kanagawa, Japan, in April 2022, and from one house located in Saitama, Japan, in July 2022 (Table 1). The sampling start date differed between days 1 and 3 in the three cases owing to the varied delivery timing of the necessary sampling equipment to the patient's house after the positive result of the rapid antigen test was reported. The environmental surfaces for sample collection were selected from sites used by the patient, such as pillows and comforters, and the others were considered because they were frequently touched by the patient or their cohabiting family members. Therefore, several sample sites differed among the three cases owing to the composition of family members, furniture, and equipment in the respective homes.

Environmental sample collection was performed from day 1 for seven consecutive days for case 1. The family of this patient consisted of four members including the patient with COVID-19, and the home was a condominium. Among the 20 sampled objects on day 1, SARS-CoV-2 RNA was detected from the patients’ comforter and mobile phone, living room sofa, and entrance doorknob at concentrations ranging from 9.3×10^4^ to 2.4×10^5^ copies/mL (Table 2). From days 2 to 4, the viral RNA was also detected in two to four samples from objects, such as the pillow, sheet, computer keyboard, and patient’s bedroom doorknob. The RT-qPCR results of all samples tested on day 5 were negative. Although three samples from the patient’s bedroom showed positive results again on day 6, viral RNA was detected in only one environmental sample on day 7. The RT-qPCR-positive samples were examined by plaque assay to detect viable viruses, but no virus was detected on cultured VeroE6/TMPRSS2 cells.

For case 2, the environmental samples were collected from a house with four family members. On day 2, when sample collection commenced, SARS-CoV-2 RNA was detected only on the patient’s comforter (Table 3). On day 3, in addition to the environmental samples near the patient, including the pillow and comforter, the doorknob, the backrest of the chair, the refrigerator in the kitchen, and the lavatory tap also showed SARS-CoV-2 RNA amplification, ranging from 6.9 × 10^4^ to 4.6 × 10^5^ copies/mL. The RT-qPCR-positive samples were confirmed on the patient’s bedsheet and the dining room doorknob on days 4 and 5. SARS-CoV-2 RNA amplification was confirmed for three samples that were collected from the patient’s bedroom, dining room, and bathroom on day 6. On days 7 and 8, only one sample each from the table and entrance doorknob, respectively, showed positive results. Among the 133 samples, 15 RT-qPCR-positive samples were examined by plaque assay. However, no viable viruses were detected in the tested environmental samples.

For case 3, environmental samples were collected from a house with four family members. With the onset of COVID-19 defined as day 0, environmental samples were collected from 26 objects for seven consecutive days starting on day 3. SARS-CoV-2 RNA was detected in 15 samples from various rooms on day 3, including the patient’s bedroom, kitchen, dining room, living room, and bathroom (Table 4). Additionally, the viral RNA was amplified from the pacifier (2.1 ×10^4^ copies/mL). The detection rate of SARS-CoV-2 RNA was 57.7%. On day 4, viral RNA (1.3×10^5^ to 2.2×10^6^ copies/mL) was detected in six samples, including the suction opening of the air purifier in the living room. The SARS-CoV-2 RNA was confirmed in one to three samples among the tested environmental samples from days 5 to 7. and the detection rate was calculated to be 3.8-11.5%. The viral RNA detection rate further increased to 19.2% on day 8. No viral RNA amplification was noted in the environmental samples on day 9 (the final day of the research period). Among the 182 samples, 32 tested positive by RT-qPCR, and a plaque assay was performed to confirm the presence of viable viruses. However, no plaque formation was noted in the VeroE6/TMPRSS2 cells from any of the tested samples.

## Discussion

This study investigated the fluctuation of environmental contamination levels of SARS-CoV-2 in the homes of housebound patients with COVID-19. The sample collection was performed in April 2022 for cases 1 and 2 when the number of new COVID-19 cases had gradually decreased to approximately 50,000 cases per day from a peak of approximately 100,000 cases per day in February 2022 during the Omicron variant wave in Japan [11]. In contrast, case 3 was assessed at the end of July 2022 when the number of new COVID-19 cases began to increase starting in early July, reaching a peak of approximately 200,000 cases per day [11]. A total of 455 environmental samples were collected from these three cases, and 63 showed a positive result by RT-qPCR, showing a detection rate of 13.8%. However, no viable viruses were detected in any of the RT-qPCR-positive samples. Although environmental contamination by the viral remnants containing SARS-CoV-2 RNA extended throughout the house, these findings illustrated that viable viruses are considered unlikely to be present on environmental surfaces.

Although the maximum detection rate of environmentally contaminated SARS-CoV-2 RNA among the sample collection days was calculated to be 57.7% on day 3 in case 3, it gradually decreased to 7.7% by day 7. However, the SARS-CoV-2 RNA detection rate increased again to 19.2% on day 8, eventually reducing to 0% in the samples collected on day 9. Similarly, an increase in the viral RNA detection rate was also observed in cases 1 and 2. The median duration of symptoms of COVID-19 for those infected with the Omicron variant was five days [12]. Therefore, environmental contamination of viral RNA may have increased as patients who had just recovered from COVID-19 resumed more of their daily living activities.

A previous study conducted in Utah in 2020, examining the frequency of household surface environmental contamination by SARS-CoV-2 [13], reported a high SARS-CoV-2 RNA positivity rate on nightstands, pillows, and light switches, ranging from 14% to 67% in 10 household COVID-19 case analyses. Another study conducted in California and Colorado, USA, from January to April 2021 in 124 households also showed a high SARS-CoV-2 RNA positivity rate in the samples collected from nightstands (44.1%) and pillows (40.9%) [14]. In that study, the viable viruses were isolated from only 0.2% of collected samples, including those from a nightstand, remote control, and kitchen counter of the same household [14]. Although plaque assays were performed on 62 samples in this study, no viable SARS-CoV-2 was observed. The SARS-CoV-2 stability on the surface of various materials has been reported under experimental conditions [15,16]. SARS-CoV-2 showed lower stability on paper, and no viable virus was detected after 3 hours of incubation at room temperature (22°C). Conversely, SARS-CoV-2 was detected for up to one day on cloth and four days on plastics [16]. The duration of viral stability on environmental surfaces is lower in the real world because it corresponds to the viral load of droplets spread during coughing and sneezing.

This study had some limitations. First, environmental samples were collected for seven consecutive days to determine the temporal change of viral contamination in the house, but only three cases were enrolled. Hence, further research with a larger sample size is warranted to confirm our results. Second, since we did not collect data on clinical symptoms, CT values of patients’ samples, and vaccination records, we could not analyze the correlation between environmental contamination levels and patient recovery. Finally, only environmental surface samples were collected in this study; air samples should also be collected and analyzed owing to the possibility of aerosol transmission.

## Conclusions

This study showed that the environmental contamination level of SARS-CoV-2 tended to fluctuate in a household setting and that virus contamination eventually disappeared after seven to nine days, even in objects used by the patient. Moreover, since we did not detect viable viruses in this study, the risk of SARS-CoV-2 transmission via fomites is expected to be relatively low. Infection control management should focus more on preventing, or at least controlling, aerosol and droplet transmission.
